# The PUF RNA-binding protein, FBF-2, maintains stem cells without binding to RNA

**DOI:** 10.1261/rna.080307.124

**Published:** 2025-05

**Authors:** Brian H. Carrick, Sarah L. Crittenden, MaryGrace Linsley, Stephany J. Costa Dos Santos, Marvin Wickens, Judith Kimble

**Affiliations:** Department of Biochemistry, University of Wisconsin-Madison, Madison, Wisconsin 53706, USA

**Keywords:** PUF RNA-binding protein, PUF partnerships, *C. elegans*, germline stem cells, sperm/oocyte cell fate decision

## Abstract

Like all canonical PUF proteins, *Caenorhabditis elegans* FBF-2 binds to specific RNAs via tripartite recognition motifs. Here, we report that an FBF-2 mutant protein that cannot bind to RNA is nonetheless biologically active and maintains stem cells. This unexpected result challenges the conventional wisdom that RBPs must bind to RNAs to achieve biological activity. Also unexpectedly, FBF-2 interactions with partner proteins can compensate for the loss of RNA binding. FBF-2 only loses biological activity when its RNA-binding and partner interactions are both defective. These findings highlight the complementary contributions of RNA-binding and protein partner interactions to the activity of an RNA-binding protein.

## INTRODUCTION

RNA-binding proteins (RBPs) pervade eukaryotic biology, from yeast to humans (Gerstberger et al. 2014). The conventional view is that RNA binding is essential for RBP biological activity (Lunde et al. 2007; Corley et al. 2020). Based on this view, mutations in RNA-binding residues are often mutated to abolish RBP activity (Siomi et al. 1994; Daigle et al. 2013).

**FIGURE 1.**
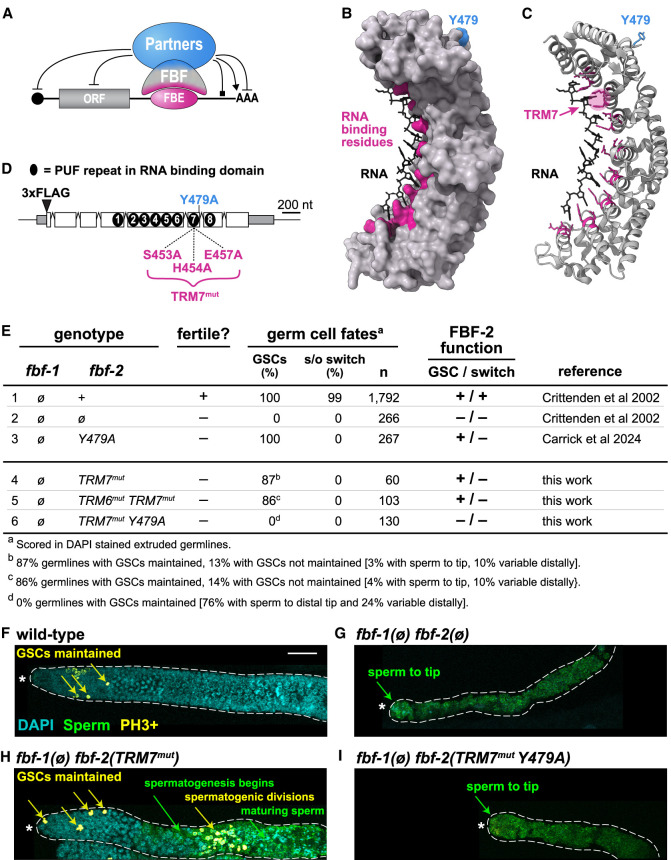
FBF-2 in vivo function requires both RNA- and protein-binding residues. (*A*) FBF binds to RNA and protein partners via distinct interfaces (magenta for RNA, blue for partners) to control various activities: RNA repression, blunted end; RNA activation, arrowhead; RNA binding, square end. (ORF) Open reading frame; (FBE) FBF-binding element. (*B*,*C*) Crystal structure of FBF-2 (*B*, surface; *C*, ribbon) binding to RNA (PDB: 3K5Y) (Wang et al. 2009). RNA-binding residues, magenta; Y479 partner interface, blue. TRMs in each PUF repeat mediate RNA binding; TRM7 is highlighted in *C*. (*D*) *fbf-2* mRNA and FBF-2 protein features. Untranslated regions (gray boxes), coding regions (white boxes), introns (peaked lines), PUF repeats (black ovals). Sites for insertion of 3xFLAG and relevant mutations are indicated. (*E*) FBF-2 TRM mutant effects on germline fates, scored in DAPI-stained extruded gonads. GSCs, percentage of germlines with stem cells maintained to the distal end; s/o switch, percentage of germlines with a successful sperm-to-oocyte switch. n, number of gonads scored. TRM7^mut^ = (S453A, H454A, E457A), TRM6^mut^= (N415A, Y416A, Q419A). (*F*–*I*) Representative z-projection images of extruded adult gonads, stained for DNA (DAPI, cyan), sperm (αSP56, green), and a cell cycle marker, phosphohistone H3 (αPH3, yellow). αPH3 marks cells in both mitotic and meiotic G2/M phase. GSC maintenance is inferred from mitotic divisions in the distal gonad (αPH3-positive staining, yellow arrows); spermatogenic meiotic divisions occur more proximally (αPH3-positive staining, green arrows), where SP56-positive staining indicates sperm differentiation. Dotted line marks gonad boundary; asterisk marks distal end. Twenty micrometers of scale bar in *F* applies to *F*–*I*.

The RNA-binding domain (RBD) of PUF (Pumilio and FBF) RBPs is defined by eight “PUF repeats” (Fig. 1A–C). These repeats form a crescent that binds to RNA on its inner surface and binds to protein partners on its outer surface (Fig. 1A–C; Zamore et al. 1997; Edwards et al. 2001; Wang et al. 2001; Campbell et al. 2012b; Friend et al. 2012; Weidmann et al. 2016; Qiu et al. 2019; Carrick et al. 2024). In vitro, RNA binding of the PUF RBP relies on tripartite recognition motifs (TRMs), which reside in each PUF repeat. TRM motifs recognize and bind to nucleotides within the PUF RNA-binding element and hence define PUF sequence specificity (Fig. 1B–D; Wang et al. 2002; Campbell et al. 2012a). However, their biological role has not yet been explored.

In addition to binding RNAs, PUF proteins also interact with proteins that help set the RNA regulatory mode, for example repression (e.g., Ccr4-Not complex) (Goldstrohm et al. 2007; Webster et al. 2019) or activation (e.g., GLD-3, GLD-2) (Suh et al. 2009) and can also affect PUF RNA-binding affinity (e.g., CPEB, Nanos, LST-1) (Fig. 1A; Campbell et al. 2012a; Weidmann et al. 2016; Qiu et al. 2019). One interaction site used by multiple protein partners occurs at the R7/R8 loop between PUF repeats 7 and 8 on the outer surface of the PUF RBD crescent (Fig. 1B,C; Campbell et al. 2012b; Wu et al. 2013; Qiu et al. 2019, 2023; Carrick et al. 2024). Within the R7/R8 loop of nematode FBF-2, a paradigmatic PUF protein, a single tyrosine (Y479) stands out as critical for binding multiple partners; indeed, its alanine substitution (Y479A) abolishes partner binding when assayed in vitro, in yeast, and in nematodes (Campbell et al. 2012b; Carrick et al. 2024).

FBF-2 drives two major biological functions in the *Caenorhabditis elegans* germline: maintenance of germline stem cells (GSCs) and promotion of the sperm-to-oocyte cell fate switch (s/o switch) (Zhang et al. 1997; Crittenden et al. 2002; Lamont et al. 2004). FBF-2 and its nearly identical counterpart FBF-1 are biologically redundant and can substitute for each other to accomplish these two functions. Thus, FBF-2 null mutants are fertile when FBF-1 is wild-type (Fig. 1E, row 1), but sterile when FBF-1 is removed, due to the loss of GSCs and failure of the s/o switch (Fig. 1E, row 2; Crittenden et al. 2002). We previously reported the molecular and biological effects of a partner defective Y479A mutant (Carrick et al. 2024). When FBF-1 was wild-type and germlines essentially normal, the FBF-2(Y479A) mutant protein changed the RNA binding, as assayed by eCLIP. That shift revealed that partner interactions modulate FBF-2 RNA-binding strength at specific FBF-binding elements (FBEs). On the other hand, when FBF-1 was gone, Y479A was able to maintain stem cells but failed to promote the s/o switch (Fig. 1E, row 3; Carrick et al. 2024). Therefore, Y479-dependent partnerships are essential for one FBF-2 biological activity but not both.

Here, we tested whether the Y479A mutant protein must bind RNA to accomplish its role in GSC maintenance. Unexpectedly, we found that loss of RNA binding on its own does not abolish FBF-2 activity and that Y479-dependent partnerships compensate for the loss of RNA binding. More broadly, our findings raise the possibility that other RBPs lacking the ability to bind RNA may nonetheless retain biological activity.

## RESULTS AND DISCUSSION

### FBF-2 RNA-binding mutants retain biological function

We predicted that the FBF-2 mutant, Y479A, would rely on RNA binding for its ability to maintain stem cells. To test that prediction, we mutated three key TRM residues in PUF repeat 7 (S453A H454A E457A, Fig. 1D) of an endogenous, FLAG-tagged, *fbf-2* gene, thus creating the TRM7^mut^ mutant. We chose these residues because any one of the three TRM7 alanine substitutions abolished FBF-2 RNA binding, when assayed in vitro or by yeast three-hybrid (Valley et al. 2012); moreover, alanine substitutions of these same TRM residues in the fly Pumilio protein were used to eliminate RNA binding in reporter assays (Weidmann and Goldstrohm 2012).

Before making TRM7^mut^ changes in the Y479A mutant, we introduced them into wild-type FBF-2 and scored for its two major biological activities, GSC maintenance and the s/o switch (see Introduction). Our expectation was that loss of RNA binding would destroy both activities and that TRM7^mut^ would behave like an *fbf-2 null* [*fbf-2(ø)*] mutant. To score FBF-2 activities, we removed FBF-1 so defects were not masked by redundancy. We examined phenotypes with DAPI (Fig. 1E) and immunostaining (Fig. 1F–I). As expected, wild-type *fbf-2(+)* maintained GSCs and promoted the s/o switch (Fig. 1E, row 1; Fig. 1F), while *fbf-2(ø)* lost both functions (Fig. 1E row 2, Fig. 1G). To our surprise, TRM7^mut^ did not behave like the null (Fig. 1E, compare rows 2 and 4; compare Fig. 1G,H). Most (87%) TRM7^mut^ adults retained GSCs, demonstrating that contrary to expectation, this mutant retains biological activity. In contrast, all (100%) TRM7^mut^ adults lost the s/o switch (Fig. 1E, line 4; Fig. 1H). The TRM7^mut^ was thus able to exert one function (GSC maintenance), but not another (s/o switch), much like Y479A (Fig. 1E, compare rows 3 and 4). Regardless, the key conclusion is that TRM7^mut^ retains biological activity—it is sufficient for GSC maintenance. To push the limits of this unexpected result, we mutated the key TRM residues in the neighboring sixth PUF repeat (TRM6: N415A, Y416A, Q419A) to create TRM6^mut^ TRM7^mut^ double mutants. However, TRM6^mut^ did not enhance the TRM7^mut^ phenotype (Fig. 1E, compare rows 4 and 5), consistent with TRM7^mut^ being sufficient to abolish RNA binding (see Fig. 3 for molecular confirmation).

### FBF-2 RNA binding and partner interactions both promote GSC self-renewal

Our original question was whether Y479A requires RNA binding to maintain GSCs. To address that question, we generated TRM7^mut^ Y479A, a double mutant that removes both RNA-binding and Y479A-dependent partner interactions. This double mutant confirmed our expectation. TRM7^mut^ Y479A mimicked an FBF-2 molecular null when assayed without FBF-1 (Fig. 1E, compare rows 2 and 6). Thus, both *fbf-2(ø)* and Y479A TRM7^mut^ mutant germlines lacked GSCs at the distal end when assayed with DAPI. This was confirmed by the absence of staining with a marker for dividing cells (Fig. 1I). Although both *fbf-2(ø)* and TRM7^mut^ Y479A mutant germlines lacked GSCs, we did find minor differences. All *fbf-2(ø)* and most (76%) Y479A TRM7^mut^ mutant germlines had mature sperm all the way to their distal end (Fig. 1G,I); however, some (24%) Y479A TRM7^mut^ germlines contained cells that had not become mature sperm distally, suggesting marginal activity in some germlines (Supplemental Fig. S1A). We speculate this residual activity may be due to partner interactions that occur outside of the R7/R8 loop (e.g., the Ccr4-Not complex or Argonaute). Regardless, no Y479A TRM7^mut^ double mutants were able to maintain GSCs. We conclude that Y479A does indeed rely on RNA binding to maintain GSCs, and that unexpectedly Y479-dependent partner interactions compensate for the loss of RNA binding for that same biological function.

### TRM7^mut^ Y479A behaves like a null when assayed in the presence of FBF-1

**FIGURE 2.**
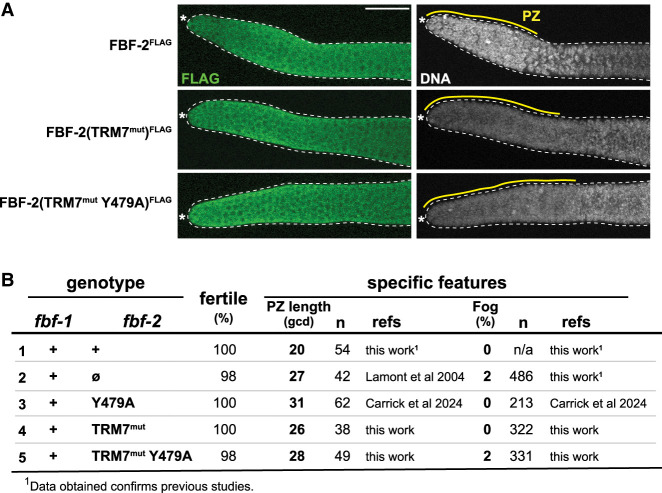
Y479A TRM7^mut^ behaves like a null when assayed in the presence of wild-type FBF-1. (*A*) Representative z-projection images of extruded adult gonads, stained for FLAG:FBF-2 (αFLAG, green) and DNA (DAPI, gray). Dotted line marks gonad boundary; asterisk marks distal end. Twenty micrometers of scale bar in the *top left* image applies to all images. Yellow line marks approximate length of the progenitor zone (PZ). (*B*) FBF-2 mutant defects in the presence of wild-type FBF-1. PZ, progenitor zone size; fertile, animals capable of producing self-progeny. All animals that were not fertile were feminized (only oocytes, Fog).

**FIGURE 3.**
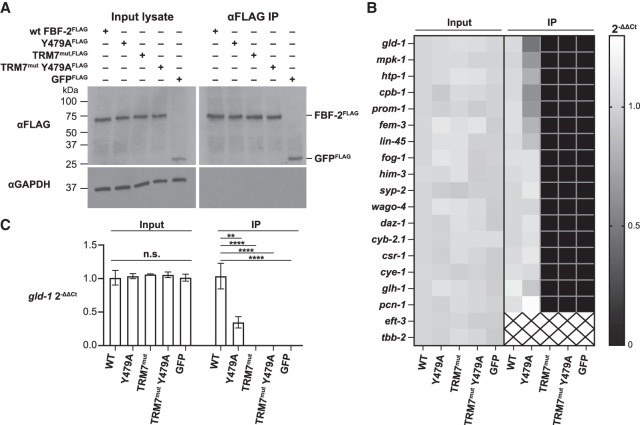
TRM7^mut^ eliminates FBF-2 binding to target RNAs in vivo. (*A*) TRM mutations do not change FBF-2 protein abundance or immunoprecipitation efficiency. Representative western blot for RIP-qPCR experiment. *Left*, input lysates (1%); *right*, FLAG IP (1%). FLAG-tagged FBF-2 variants and GFP are immunoprecipitated; negative control (GAPDH) is not immunoprecipitated. (*B*) TRM mutations abolish FBF-2 mRNA binding. Heatmap depicts results from quantitative PCR of FBF target mRNAs and control mRNAs after αFLAG IP, using 3xFLAG::FBF-2 for variants and 3xFLAG::GFP for the control. Mean mRNA abundance in input (*left*) and IPs (*right*) was calculated with the comparative *C*_*T*_ method $\left(2^{-\Delta \Delta C_{T}}\right)$ (Schmittgen and Livak 2008), using *rps-25* for normalization and making all comparisons to the wild-type sample. $2^{-\Delta \Delta C_{T}}$= 1, no change in mRNA level compared to wild-type; $2^{-\Delta \Delta C_{T}}$< 1, less mRNA than wild-type; $2^{-\Delta \Delta C_{T}}$> 1 more mRNA than wild-type (gray level scale indicated to *right* of heatmap). Because no specific signal was seen for negative controls *eft-3* and *tbb-2* in the wild-type IP sample, $2^{-\Delta \Delta C_{T}}$ was not calculated (boxes containing black X). (*C*) Effect of FBF-2 mutations on binding to *gld-1* RNA. Example bar graph of $2^{-\Delta \Delta C_{T}}$ values for one of the target RNAs tested, *gld-1*. No significant differences in *gld-1* levels were seen in the input. The *gld-1* RNA abundance is significantly different in immunoprecipitated samples. (**) *P* = 0.0017, (****) *P* < 0.0001.

We next investigated TRM7^mut^ and TRM7^mut^ Y479A in the presence of wild-type FBF-1, because these germlines were essentially normal, allowing us to test for effects on protein stability and cellular distribution, and because other *fbf-2* mutants had minor defects in this situation (see below). By immunostaining, both the levels and distribution of TRM7^mut^ and TRM7^mut^ Y479A mutant proteins were comparable to wild-type (Fig. 2A, also see Fig. 3A). Therefore, any changes are likely due to an effect on activity rather than stability.

Previous work identified minor defects of *fbf-2* mutants when assayed in the presence of wild-type FBF-1. First was a lengthening of the progenitor zone (PZ), a region in the distal germline that includes GSCs and GSC daughters that have just begun to differentiate. PZ length increased from ∼20 germ cell diameters (gcd) in wild-type animals to >25 gcd in both *fbf-1(+) fbf-2(*ø*)* and *fbf-1(+) fbf-2(*Y479A*)* (Fig. 2B, rows 1–3; Lamont et al. 2004; Carrick et al. 2024). Second was a low penetrance Fog (Feminization of germline) defect: 2% Fog in *fbf-1(+) fbf-2(*ø*)* compared to 0% Fog in wild-type (Fig. 2B, rows 1 and 2); this low penetrance Fog was not seen for Y479A (Fig. 2, row 3; Carrick et al. 2024).

For the TRM7^mut^ single and TRM7^mut^ Y479A double mutants, PZ length increased (Fig. 2B, rows 4 and 5), much like for FBF-2 null and Y479A mutants (Fig. 2A, rows 2 and 3). In contrast, TRM7^mut^ or Y479A single mutants had no feminized Fog germlines (Fig. 2B, rows 3 and 4), like wild-type, but TRM7^mut^ Y479A double mutants did generate 2% Fog germlines (Fig. 2B, row 5), like FBF-2 null (Fig. 2B, row 2). We conclude that the activity of the TRM7^mut^ Y479A double mutant is comparable to that of an FBF-2 null, both in the presence of FBF-1 (Fig. 2B) and in the absence of FBF-1 (Fig. 1E).

### TRM7^mut^ abrogates RNA binding in vivo

To test whether TRM7^mut^ abolishes RNA-binding activity in nematodes*,* we used RNA immunoprecipitation followed by quantitative PCR (RIP-qPCR) to assess FBF-2 binding to RNA targets in vivo. Importantly, these experiments were done using animals with normally organized and functional germlines due to the presence of wild-type FBF-1 (Fig. 2). We performed three biological replicates for each of five FLAG-tagged proteins: wild-type FBF-2, Y479A, TRM7^mut^, TRM7^mut^ Y479A, and GFP as a negative control (Fig. 3A). These FLAG-tagged proteins were expressed at comparable levels and immunoprecipitated with similar efficiency (Fig. 3A). After extracting RNAs that coimmunoprecipitated with the FLAG-tagged proteins and converting them to cDNA, we employed qPCR to probe for 20 different RNAs, including 17 FBF-2 target RNAs and two nontarget RNAs (*eft-3* and *tbb-2*). We used another nontarget RNA (*rps-25*) for normalization using the comparative C_T_ method (Schmittgen and Livak 2008). Each biological replicate was assessed in technical triplicate, and RNA levels for each mutant were compared to wild-type (Supplemental Table S1). Input RNA abundance was similar for all samples (Fig. 3B, input columns).

These IPs confirmed results with wild-type FBF-2 and Y479A, assayed previously by eCLIP (Carrick et al. 2024). Thus, wild-type FBF-2 and Y479A bound to all 17 known targets but not to the nontarget RNAs (Fig. 3B, IP WT and Y479A columns), and Y479A binding was weaker than wild-type for specific targets (e.g., *gld-1*, Fig. 3B, IP Y479A column; Fig. 3C). More importantly, the IPs demonstrated that TRM7^mut^ and TRM7^mut^ Y479A proteins did not bind RNA (Fig. 3B, IP TRM7^mut^ and TRM7^mut^ Y479A columns), much like the GFP negative control (Fig. 3B). Figure 3C quantitates results for binding to the *gld-1* target RNA and shows that abundance differences of immunoprecipitated RNA is significant. We conclude that the TRM7^mut^ destroys RNA binding in vivo.

### Conclusions and implications

This work investigates the in vivo significance of key RNA-binding residues in FBF-2, a paradigmatic PUF protein. Our results lead to three major conclusions. First, RNA binding is not essential for one FBF-2-dependent biological function, maintenance of GSCs: most TRM7^mut^ mutants maintain GSCs despite a lack of RNA binding. This unexpected result is important because it challenges the conventional wisdom that RBPs must bind to RNAs to exert biological activity. Second and in contrast to the first conclusion, RNA binding is required for the sperm-to-oocyte cell fate switch. The differing first and second conclusions—RNA binding required for one activity but not the other—highlight the likelihood of distinct mechanisms for the two major biological activities of a single PUF protein. Third, FBF-2 partner interactions are essential for GSC maintenance in TRM7^mut^ mutants, a conclusion that highlights the importance of PUF partner interactions and provides a clue about how an RNA-binding defective RBP may nonetheless retain biological function.

**FIGURE 4.**
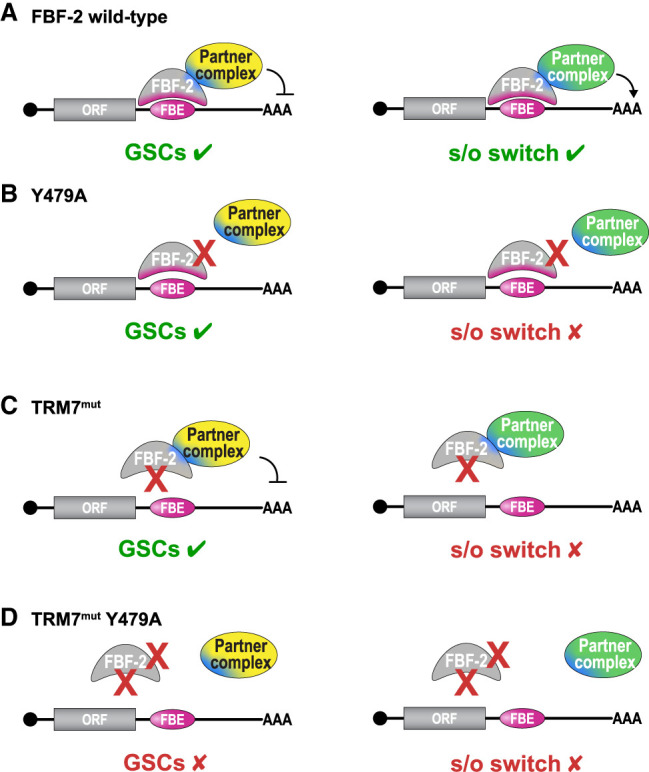
Models for effects of RNA binding and protein partner on FBF-2 function. (*A*–*D*) Models to illustrate how RNA binding and protein partner interactions modulate FBF-2 function. Target RNAs (straight lines) with a cap (circle) at 5′ end, open reading frame (ORF), and 3′ untranslated region (3′ UTR) containing an FBF-binding element (FBE). (GSCs) Germline stem cells; (s/o switch) sperm/oocyte fate switch. (*A*) Wild-type FBF-2 binds to an FBE in its target RNA and also interacts with protein partner complexes. These two binding interactions are sufficient for both GSC self-renewal (*left*) and the s/o switch (*right*). Both GSC-promoting (yellow) and s/o switch- promoting (green) partner complexes interact via the R7/R8 loop interface and Y479 (blue). (*B*) The FBF-2 mutant protein, Y479A, does not bind to R7/R8 loop-dependent partner complexes, and its binding to certain RNAs is affected. Y479A cannot execute the s/o cell fate switch but remains capable of GSC self-renewal. See Carrick et al. (2024) for details. (*C*) The FBF-2 mutant protein, TRM7^mut^, does not bind an FBE but does bind R7/R8 loop-dependent protein partner complexes. TRM7^mut^ remains capable of GSC self-renewal (*left*) but not for the s/o switch (*right*). See text for further explanation. (*D*) TRM7^mut^ Y479A double mutant no longer binds RNA or protein partners and loses its ability to accomplish either germline function.

Figure 4 proposes a model to illustrate our thinking about how RNA recognition and protein partnerships may work together to accomplish in vivo FBF-2 functions. Central to this model is the idea that FBF-2 interacts with distinct Y479A-dependent partner complexes to achieve its two different biological functions. Figure 4A depicts wild-type FBF-2 binding to FBEs in its target RNAs as well as to Y479-dependent partners that modulate regulatory activity. The Y479-dependent partners chosen for illustration postulate one complex that represses RNAs in GSCs and a different complex that activates RNAs to promote the s/o switch. Although these examples are consistent with available evidence, other complexes and other factors may well contribute to these fate decisions. Figure 4B depicts Y479A unable to bind R7/R8 loop-dependent partners, and affecting binding to certain RNAs, leading to the loss of the s/o fate switch, but not to the loss of GSC self-renewal (Carrick et al. 2024). Figure 4C depicts TRM7^mut^ protein binding to partners but not to FBEs. We suggest that GSC-promoting partner complexes regulate RNAs without FBF-2 RNA binding (Fig. 4C, left), but that the switch-promoting partner complex requires FBF-2 RNA binding (Fig. 4C, right). The challenge now is to understand how a GSC-promoting partner complex operates in the absence of FBF-2 RNA binding. One simple model invokes the involvement of a different RBP that brings TRM7^mut^ protein to target RNAs (Qiu et al. 2025). Alternatively, an unknown RNA-independent role may be responsible. Figure 4D depicts the TRM7^mut^ Y479A double mutant protein, that no longer binds to either RNA or Y479-dependent partners and that no longer has biological activity. Our model thus proposes that FBF-2 operates via a multilayered regulatory mechanism, where different mechanisms compensate for one another, ensuring that essential biological processes are maintained even when one mode of interaction is compromised or downregulated. Such a multilayered mechanism may hold true for other PUF proteins and indeed other RBPs.

PUF proteins, and RBPs more generally, are implicated in a wide spectrum of human diseases (Gennarino et al. 2015; Kapeli et al. 2017; Gennarino et al. 2018; Choi and Thomas-Tikhonenko 2021; Prashad and Gopal 2021). Disease-associated mutations can occur not only in RNA-binding residues, but also in residues located outside the RBD, many within regions involved in protein–protein interactions. Our findings emphasize the critical need to understand how both RNA recognition and protein partnerships influence RBP function in vivo. While it might seem intuitive that a mutation disrupting RNA binding would eliminate an RBP's biological activity, our work shows that this assumption is oversimplified. Consistent with that idea, RBPs with mutations in RNA-binding residues can be oncogenic (Choi and Thomas-Tikhonenko 2021), a phenomenon that may rely on interactions with protein partners. A deeper understanding of RNA regulatory mechanisms is essential for unraveling the complexities of disease pathology and developing effective therapeutic strategies. Together, our work highlights the importance of studying RBP function in the context of its binding partners in addition to its RNA binding to target RNAs.

## MATERIALS AND METHODS

### *C. elegans* maintenance

*C. elegans* were maintained on NGM seeded with OP50 with standard techniques and grown at 20°C (Brenner 1974). Hermaphrodite animals were grown to 24 h past the L4 stage unless otherwise noted. Strains used are listed in Supplemental Table S2.

### CRIPSR–Cas9-mediated gene editing

New alleles were created by co-CRISPR editing using a CRISPR/Cas9 RNA–protein complex (Arribere et al. 2014; Paix et al. 2015; Dokshin et al. 2018). Animals were injected with a mix containing a gene-specific crRNA (5 μM, IDT-Alt-R), unc-58 crRNA (4 μM, IDT-Alt-R), tracRNA (4.5 μM, IDT), unc-58 repair oligo (1 μM, IDT), gene-specific repair oligo (5 μM, IDT), and Cas9 protein (3 μM, glycerol free, IDT). F1 progeny of injected hermaphrodites were screened for edits by PCR, homozygosed, sequenced and outcrossed against wild-type prior to analysis. See Supplemental Table S3 for guide RNA and repair template sequences.

### mos1-mediated single-copy insertion (mosSCI)

DNA encoding *mex-5* promoter: eGFP with introns: 3xFLAG: *tbb-1* 3′UTR: *gpd-2* SL2 splice site: mCherry with introns: 3xmyc: PGL-1 RGG repeat: *tbb-1* intergenic region was cloned into pCFJ151 to create pJK1728. The transgene was inserted into the *ttTi5605* site on *LGII* of strain EG6699 using the *mos1-*mediated single-copy insertion method to generate *qSi100* (Frokjaer-Jensen et al. 2008). The presence of the transgene was verified by PCR and Sanger sequencing.

### Phenotypic analysis

Adult animals were scored as fertile or sterile using a dissecting scope. Sterile animals were then mounted on agarose pads and scored for germ cell morphology on a compound microscope. PZ length in gcd was scored in DAPI-stained animals by counting gcd from the distal tip of the germline to the start of meiotic entry (Crittenden et al. 2023). Cells at the distal end of DAPI-stained gonads were scored as GSCs, sperm, or variable. GSC germlines had a PZ (appropriately sized cells followed by crescent-shaped nuclei characteristic of early meiotic prophase). Sperm was identified by distinctive highly condensed DNA. “Variable” gonads contained enlarged nuclei, crescents, or ambiguous cells at the distal end.

### RIP-qPCR

Strains JK5081, JK5810, JK5984, JK6593, and JK6737 were cultivated at 20°C and grown to early adulthood (24 h after L4) in all RIP-qPCR replicates. The developmental stage was evaluated with a Leica Wild M3Z stereoscope to score body size and stage-specific marks (e.g., vulva formation). Animals were kept on standard NGM plates and fed *Escherichia coli* OP50 as previously described (Stiernagle 2006). Age-synchronized first stage larvae (L1) were obtained by bleach synchronizing gravid adults by standard methods (Lewis and Fleming 1995). Briefly, gravid adults were treated with 2:1 bleach:4N NaOH to isolate embryos. Embryos were resuspended in M9 buffer (per 1 L of buffer: 6 g Na_2_HPO_4_, 3 g KH_2_PO_4_, 5 g NaCl, 1 mL of 1 M MgSO_4_) without food in a ventilated Erlenmeyer flask at 20°C for 20 h. L1s were pelleted at 2500 rcf for 2 min, washed twice with 15 mL of M9, and distributed to 10 cm NGM plates preequilibrated to 20°C. Plates were preseeded with 1.5 mL of 40× concentrated OP50. Three biological replicates of each genotype were obtained. At least 100,000 animals were used per replicate, and each plate contained no more than 10,000 worms per plate.

Once animals reached the L4 + 24-h stage, live worms were quickly rinsed from plates into a 15 mL falcon tube with cold M9 + 0.01% Tween-20 (M9Tw), washed once with cold M9Tw, pelleted at 200 RCF in cold M9Tw, and transferred by glass pipet to a 2 mL tube, and snap frozen in liquid nitrogen. Pellets were stored at −80°C.

Pellets were thawed by adding 800 μL ice-cold lysis buffer (50 mM HEPES pH 7.5, 100 mM NaCl, 1% NP-40, 0.1% SDS, 0.5% sodium deoxycholate, 1× Roche cOmplete, EDTA-free protease inhibitor cocktail, 1 U/μL SUPERase•In RNase inhibitor) and rocking for 20 min at 4°C. Thawed pellets were centrifuged at 1000 RCF at 4°C for 1 min and washed three times with 800 μL cold lysis buffer. One milliliter of lysis buffer was added to the pellet along with a 5-mm stainless steel ball (Retsch). Lysis was performed at 4°C with a Retsch 400 MM mill mixer (3× 10-min cycles of 30 Hz). Cracking of tube lid was prevented by adding two small pieces of duct tape to the lid just prior to lysis. Complete tissue lysis was confirmed by observing a small aliquot of lysate at 40× magnification. Lysate was clarified at 16,000 RCF for 15 min at 4°C. Protein concentration was determined using Bio-Rad Protein Assay Dye (Bio-Rad 5000006) and measuring absorbance at 595 nm on a Bio-Rad SmartSpec 3000.

To prepare antibody conjugated beads, 10 μg mouse αFLAG was incubated with 4.5 mg protein G Dynabeads (Novex, Life Technologies, 10003D) for 60 min at RT. Beads were then washed 2× with lysis buffer. Twenty milligrams of total protein was incubated with the antibody-bead mixture for 4 h at 4°C. Beads were washed three times with lysis buffer and then three times with wash buffer (same as lysis but with 500 mM NaCl). Successful IP was confirmed by analyzing 1% of elution by western blot. 1% of beads were resuspended in (2% [w/v] SDS, 0.1% βME, 10% glycerol, 50 mM Tris pH 8), incubated for 10 min at 100°C and analyzed by SDS-PAGE (4%–20% acrylamide gel). For primary antibodies, blots were incubated overnight at 4°C at the following dilutions: αFLAG M2 (1:1000; Sigma-Aldrich F1804), αGAPDH (1:10,000; Proteintech 60004-1-Ig). For secondary antibody, blots were incubated for 1 h at RT with HRP-conjugated anti-mouse (1:10,000, Jackson ImmunoResearch 115-035-003).

RNA was purified by adding 500 μL acid-phenol:chloroform:isoamyl alcohol (125:24:1, Invitrogen AM9722, PCA) to the remaining beads (still in last wash). Samples were mixed by gentle shaking and were separated by centrifugation for 15 min at 15,000 rpm at 4°C. The aqueous layer was removed (∼500 μL) and further extracted by three additional extractions (1× PCA followed by 2× chloroform:isoamyl alcohol). After the extractions, the aqueous layer was removed and ∼1 mL of 100% ethanol was added to the samples, which were gently mixed and incubated at −50°C for >1 h. RNA was pelleted by centrifugation for 30 min at 15,000 rpm at 4°C. Pellets were washed once with ∼70% ethanol and resuspended in 43 μL of water. Eight units of TURBO DNase (Life Technologies AM2238) was then added for 1 h at 37°C. RNA was purified using the GeneJet RNA Purification kit (Thermo Fisher Scientific K0732) and eluted in 30 μL of water. RNA samples were stored at −80°C until use.

RNA was converted to cDNA with SuperScript III First-Strand Synthesis System (Invitrogen 18080051) using random hexamers as primers. Quantitative PCR was carried out in technical triplicate in a Roche LightCycler 480 using the LightCycler 480 SYBR Green I Master (Roche 04887352001). Average *C*_*T*_ of the technical replicates for each biological replicate is given in Supplemental Table S1. Primers used for each gene tested are listed in Supplemental Table S4. The comparative *C*_*T*_ method $\left(2^{-\Delta \Delta C_{T}}\right)$ was used to calculate relative amounts of RNA present using *rps-25* to normalize and making all comparisons to wild-type (Schmittgen and Livak 2008). Significance was calculated in GraphPad Prism 10.0.0 using one-way ANOVA and Dunnett's multiple comparisons test (all compared to wild-type).

### Immunostaining and imaging

Animals were staged at mid-L4 and grown for 24 h at 20°C and then processed for immunostaining. We immunostained gonads as described with minor modifications (Crittenden et al. 2023). Gonads were dissected in PBS containing 0.1% (v/v) Tween-20 (PBST) and 0.25 mM levamisole. Gonads were fixed in 4% (w/v) paraformaldehyde in PBST for 10 min, then permeabilized in 0.2% (v/v) Triton-X in PBST. Next, gonads were incubated for at least 30 min in a blocking solution (30% goat serum in PBST), washed three times with PBST, and incubated overnight at 4° with primary antibodies diluted in the blocking solution. After washing, secondary antibodies were diluted in blocking solution and incubated with samples for at least 1 h. To visualize DNA, DAPI was included with the secondary antibody at a final concentration of 1 ng/μL. After washing, samples were mounted in ProLong Gold (P36930; Thermo Fisher Scientific) and cured overnight to several days before imaging. All steps were performed at room temperature unless otherwise indicated. Antibody concentrations were as follows: αFLAG M2 (1:1000; Sigma-Aldrich F1804), αSP56 (1:100; Sam Ward [Ward et al. 1986]), αPH3 (1:1000, Cell Signaling Technology 9706), αMouse-Alexa647 (1:1000; Molecular Probes/Invitrogen A-31571), and αRabbit-Alexa488 (1:1000; Molecular Probes/Invitrogen A-21206). Imaging was performed on a Leica SP8 confocal microscope.

## SUPPLEMENTAL MATERIAL

Supplemental material is available for this article.

## Figures and Tables

**Brian H. Carrick:**
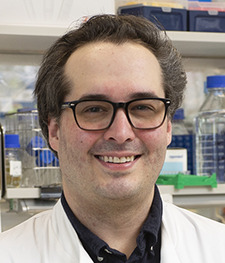


## MEET THE FIRST AUTHOR

***Meet the First Author(s)* is an editorial feature within *RNA*, in which the first author(s) of research-based papers in each issue have the opportunity to introduce themselves and their work to readers of *RNA* and the RNA research community. Brian H. Carrick is the first author of this paper, “The PUF RNA-binding protein, FBF-2, maintains stem cells without binding to RNA.” During this work, Brian was a PhD student with Judith Kimble and Marvin Wickens at the University of Wisconsin-Madison. Since then, he has moved to Cambridge, England for postdoctoral work with Lori Passmore at the MRC Laboratory of Molecular Biology. Broadly, he is interested in posttranscriptional RNA control by RNA-binding proteins, and how these proteins regulate development.**

**What are the major results described in your paper and how do they impact this branch of the field?**

The major, and quite surprising, finding of our work is that an RNA-binding protein (RBP) can maintain function when its ability to bind RNA is impaired. We discovered that FBF-2, a PUF-family RBP in *C. elegans*, can maintain germline stem cells even when its ability to bind RNA is abolished. Instead, its interactions with protein partners can compensate for the loss of RNA binding, demonstrating a previously unappreciated level of flexibility in RBP function. However, RNA binding remains essential for a separate function—regulating the sperm-to-oocyte cell fate switch—highlighting distinct mechanisms for different biological roles of the same protein. We think these results have broad implications for the field of RNA biology and suggest that RBPs can function through alternative, protein-mediated mechanisms when RNA binding is removed.

**What led you to study RNA or this aspect of RNA science?**

As an undergraduate at the University of Michigan, I had the opportunity to join the Michigan Research Community and work in David Engelke's lab as a freshman and through the rest of my undergraduate studies. He introduced me to RNA research and the vibrant RNA community, sparking my enthusiasm for the field. This experience motivated me to pursue a PhD in a program with a strong tradition in RNA science. Joining Marv and Judith's labs further solidified my passion for understanding how RNA regulates essential biological decisions.

**During the course of these experiments, were there any surprising results or particular difficulties that altered your thinking and subsequent focus?**

This project actually began with an unexpected result from a control experiment. We were mutating a protein–protein interaction interface and initially assumed that the resulting phenotype was due to the protein still binding RNA. However, when we introduced a mutation that specifically eliminated RNA binding, we were surprised to find that the protein retained partial function. We had predicted that an RNA-binding protein would lose all biological activity if it could no longer bind RNA, but this result challenged that assumption. It ultimately led to this study, where we characterized the mutant in detail and demonstrated that, even without RNA binding, the protein can still function through its interactions with other proteins.

**Are there specific individuals or groups who have influenced your philosophy or approach to science?**

I've been fortunate to learn from and be mentored by incredible scientists throughout my career. It began with Dave Engelke and his lab, where I first developed my approach to research. This continued during my graduate work with Judith and Marv, alongside the outstanding members of their groups and collaborators like Michael Sheets, Sunduz Keles, and Traci Hall. There are too many individuals to name (special shoutout to the Pizza Crew), but each has played a role in shaping how I think about science and approach research.

**What are your subsequent near- or long-term career plans?**

In Lori Passmore's group, I will be using structural, biophysical, and genomic approaches to study how poly(A) tails are controlled. After my postdoc work with Lori, I hope to establish an independent research program using multidisciplinary approaches to study the roles of RNA binding proteins in 3′ end control and during development.
